# A cohort study on longitudinal changes in postural balance during the first year after stroke

**DOI:** 10.1186/s12883-022-02851-7

**Published:** 2022-08-30

**Authors:** Dongni Buvarp, Lena Rafsten, Tamar Abzhandadze, Katharina S. Sunnerhagen

**Affiliations:** 1 Rehabilitation Medicine Research Group, Department of Clinical Neuroscience, Institute of Neuroscience and Physiology, University of Gothenburg, Per Dubbsgatan 14, 40530 Gothenburg, Sweden; 2 Sahlgrenska University Hospital, Gothenburg, Sweden

**Keywords:** Cerebrovascular Accident, Berg Balance Scale, Longitudinal Analysis, Impairment of Postural Balance, Stroke Recovery

## Abstract

**Introduction:**

Many patients with strokes report increased incidence of fall that can be due to impaired postural balance. The recovery of balance in patients with varying degrees of impairments and activity limitations is less studied, and whether individuals with mild paresis can recover their balance faster is unclear. Better knowledge about factors influencing the recovery of postural balance can be used to guide clinical management after stroke to provide the right rehabilitation to the right person at the right time, and thus to avoid potential fall incidences.

**Objective:**

This study aims to examine longitudinal changes in postural balance during the first year after stroke.

**Methods:**

Postural balance was assessed using the Berg Balance Scale (BBS) within 5 days, 1, 2, and 3 months and 1-year post-stroke. Stroke severity was stratified using a cluster analysis by including multidimensional baseline measures. A longitudinal mixed-effect model was constructed to analyze changes in proportional balance impairment by stroke severity over time. Individuals with a cut-off of BBS below 45 scores were identified through a classification algorithm using baseline predictors.

**Results:**

A total of 135 patients were stratified to mild stroke (77 [57%] patients) or moderate stroke (58 [43%] patients). Ninety-three patients were included in the longitudinal analysis. Significant recovery was found at 1-year for moderate stroke (48% recovery from the initial impaired postural balance, adjusted *P* < 0.001), but not for mild stroke, after adjusting for age and cognition. Both stroke severities had a maximal recovery in postural balance at 3 months post-stroke, but the moderate stroke group deteriorated after that. Patients with higher age and worse cognition had more severe balance impairments. The classification model achieved a sensitivity of 0.95 (95% confidence interval [CI]: 0.91–0.98) and a specificity of 0.99 (95% CI: 0.98–1.0) for classifying individuals with BBS below 45 points.

**Conclusions:**

This study indicates that continuous improvements in postural balance ends at 3 months regardless for mild or moderate stroke groups, and patients with moderate stroke significantly deteriorate in postural balance after 3 months.

## Introduction

Stroke survivors experience many types of long-term consequences. Impaired postural balance is one of the well-recognized residual impairments in patients after stroke, and is often associated with social isolation and limited mobility, resulting in a decline in the quality of life [1, 2]. A majority of stroke survivors report a history of fall incidents during the first year after stroke due to impairment in postural balance, which has led to a high incidence of fall-related injuries and mortality [3].

The severity of impaired postural balance after stroke is generally related to higher age, impaired motor function and cognitive deficits [4, 5]. An improvement in function may occur within the first few weeks after stroke, and may be attributable to the combination of spontaneous recovery and the effect of rehabilitation [6]. However, for a substantial number of patients, the continuous gain in recovery seems to diminish 3 months after stroke onset [7]. A decline may then occur in patients with more severe initial impairments [8]. Little is known about whether the recovery of postural balance follows a similar longitudinal pattern as shown in other impairments, such as functional mobility in stroke [8], and whether the postural balance recovery rate differs between stroke severity.

Knowledge about longitudinal progression in impairment of postural balance after stroke is a pre-requisite to understanding the need for appropriate mobility aids and early balance training. This could greatly contribute to identifying individuals who have residual balance impairment and may be susceptible to a high risk of falling. Early identification of patients with potential balance impairment would also allow interventions for potential falls, and significantly reduce the psychological burden for patients with stroke and next of kin [9]. The postural evaluation was commonly assessed using the Berg Balance Scale (BBS) that is a clinical tool to assess both dynamic and static balance. The BBS is a sufficient clinical screening tool to determine a risk of falling in terms of good sensitivity and reliability, which is not require extensive resource and time to conduct. A BBS score of less than 45 is a generalized cut-off score that is well-recognized in clinical practice and has previously demonstrated that patients with a lower BBS 45 more likely to fall than were those who were above the score prone to a greater risk of falling [10, 11].

The primary aim of the study was to examine longitudinal changes in postural balance between different stroke severities during the first year after stroke. The secondary aim was to identify individuals, regardless of stroke severity, who have a BBS score below 45 which is considered to be susceptible to a risk of falling.

## Methods

### Study population and design

The participants in this longitudinal and prospective study were enrolled in the Gothenburg Very Early Supported Discharge clinical trial (URL: http://www.clinicaltrials.gov. Unique identifier: NCT01622205) at Sahlgrenska University Hospital, Sweden, from September 2011 to April 2016 [12]. The GOTVED study is a randomized controlled study were 140 included patients were randomized to very early supported discharge with continued rehabilitation in the patient’s home or to a control group receiving ordinary rehabilitation. Additional information about GOTVED can be found elsewhere [12]. The study was approved by the Regional Ethical Review Board in Gothenburg (registration number:426–05 and 042–11) and was conducted in agreement with the Declaration of Helsinki. The inclusion criteria were age > 18 years; a diagnosis of ischemic or hemorrhagic stroke confirmed according to World Health Organization criteria [13]; a National Institute of Health Stroke Scale (NIHSS) score of 0–16 points, which corresponds to mild-to-moderate stroke; a Barthel Index (BI) score of 50 points or more on day 2; and a Montreal Cognitive Assessment index of 26 points or less if BI = 100. Patients with a life expectancy < 1 year (e.g., with severe malignancy) or who could neither speak nor communicate in Swedish prior to stroke were excluded. In this longitudinal and prospective study, the data were extracted from the Gothenburg Very Early Supported Discharge clinical trial, and the 140 patients are pooled into one group. Details of the full inclusion and exclusion criteria of the study trial as well as the power calculation of the study sample size were previously reported [12]. All participants provided written informed consent prior to the longitudinal trial.

### Clinical assessments

The BBS was used to assess postural balance across 5 time intervals as following: within 5 days after stroke onset (referred as baseline), within 1, 2, and 3 months post-stroke, and at 1-year after stroke. The BBS is a 14-item scale, and each item consists of five ordinal responses to assess static and dynamic balance. Static balance is defined as the ability to maintain an upright posture and the centre of mass is over the base of support [14]. Dynamic balance is defined as the ability maintain a stable base of support while completing weight shifting movements [15]. The maximum total score is 56 points (higher indicates better postural balance) [16]. A BBS score below a cut-off of 45 points indicates patients with a high risk of falling [10, 17]. The BBS scale has proven to be reliable and valid for assessing patients with acute and chronic stroke [10, 18].

Other assessments that describe the consequences of a stroke, such as impairments and activity limitations, were also performed. Overall disability post-stroke was assessed using the modified Rankin Scale (mRS) with an ordinal scale ranging from 0 to 6 in which 0 corresponds with no disability at all, 5 indicates severe disability, and 6 represents death [19]. The National Institutes of Health Stroke Scale was used to assess neurological deficit at 2 days after admission by a stroke-physician [20]. The 10-item ordinal Barthel Index (BI) was used to measure dependency in daily activities, with a score ranging from 0 to 100 (lower indicates higher dependency) [21]. Motor-sensory function in the extremities was assessed using the Fugl-Meyer Assessment Scale (FMA; lower extremity [-LE] and upper extremity [-UE]), with a lower FMA score indicating more severe impairment of function [22]. Cognitive function was screened using the Montreal Cognitive Assessment (MoCA), scored from 0 to 30 (lower indicates worse cognition) [23]. BI and MoCA were administered by occupational therapists 36–48 h after arrival at the stroke unit. An experienced and blinded physiotherapist not working at the stroke unit performed the clinical assessments. The Hospital Anxiety and Depression Scale (HADS) self-assessment questionnaire was used to assess psychological distress [24]. The 14-item questionnaire consists of two subscales, one 7-item subscale was used for assessing anxiety and the other 7-item subscale to assess depression. A total score above 7 points on a subscale of HADS (each item scored from 0 to 3) was considered to indicate symptoms of mild and moderate anxiety or depression [24].

NIHSS and MoCA were conducted as earlier as possible, whereas HADS and SIS were gathered on Day 5 to avoid extra burden for patients with stroke in their stroke care at the hospital.

### Statistical analyses

#### Baseline clustering of stroke severity

To classify stroke severity, a baseline cluster analysis was conducted to identify homogenous subgroups based on similar clinical characteristics. Multidimensional clinical variables were included, covering impairments and activity limitations that describe overall functioning and disability. The purpose for cluster analysis was not to have use pre-defined cut offs for assessment of stroke severity but to in an open and non-prejudiced way include all possible variables in the analysis. This minimizes the risk of missing factors that are there but that we haven´t considered. The dissimilarity between observations across individuals was calculated using a general dissimilarity coefficient that can handle mixed-type variables by assigning different distance measures to continuous, ordinal and nominal variables [25]. A partitioning around medoids algorithm was then used to cluster the established dissimilarity matrix, and an optimal number of clusters was determined and selected on the basis of silhouette width [26]. Internal validation and stability of clusters were evaluated further [27, 28].

To compare clinical characteristics across each cluster, either Fisher’s exact test, Pearson χ^2^, Cochran–Armitage test, Mann–Whitney *U* test or independent *t* tests was used for post hoc comparison, as appropriate. Imputation for mixed-type missing data (2.6% of the total data) was performed as previously described [8].

#### Longitudinal changes in postural balance

Patients were considered lost to follow-up and excluded from the longitudinal analysis if two or more visits were missed and/or they had more than 30% missing data in outcomes.

Considering the ordinal nature of BBS ratings, a proportional impairment of postural balance was calculated as the outcome for determining potential recovery. A longitudinal beta regression mixed-effect model was therefore appropriate for analyzing proportional data to increase clinical interpretation while avoiding shortcomings in conventional regression approaches for bounded outcomes [29]. Impairment of postural balance was defined as the difference between the maximum balance score (BBS 56 scores) and the residual balance function. The proportion of balance impairment was then equivalent to balance impairment over the maximum balance scores. The proportions were converted to an interval of 0 to 1 on a continuous scale, with an upper and lower limited bound of 0.005 and 0.995, respectively.

A multilevel longitudinal mixed-effect model was applied to analyze the changes in proportional impairment of postural balance over time across different stroke severities [29, 30]. Age, cognition, time, stroke severity, and interaction between stroke severity and time were included as fixed effects. The random intercept for each patient was also included*. P* values for multiple comparisons were adjusted using Holm-Bonferroni corrections. A two tailed significance level was defined as *P* < 0.05.

#### Classification for individuals who had a BBS score below 45.

Random forest is a robust binary classification algorithm for generating a majority vote among trees on the basis of multiple independent decision trees [31]. A random forest model was constructed for classification by using multidimensional baseline measures as predictors for classifying individuals who had a BBS score, at any point, lower than 45 during the first year of stroke which corresponded to an increased risk of falling. This was done by using multidimensional baseline measures as predictors for classifying individuals who had a BBS score, at any point, lower than 45 during the first year of stroke [32]. Tuning parameters was conducted with fivefold cross-validation, and the importance of variables was determined by the mean decrease in accuracy consequent to the permutation of each variable. The predictive performance was determined in terms of classification accuracy, sensitivity and specificity.

## Results

A total of 135 patients were eligible for the baseline analysis (median age 76 years, range 37–96, 52 females [39%], Table 1). Forty-two patients were excluded prior to the longitudinal analysis for reasons of loss to follow-up (*n* = 18), withdrawal (*n* = 18), a second stroke or other diseases that impaired motor function (*n* = 6). The differences were not statistically significant in age, sex and neurological deficits between the excluded patients and the patients included in the longitudinal analysis.

**Table 1:** Included variables for the baseline cluster evaluation and group characteristics at baseline and for longitudinal analysis

Characteristic	All (*n* = 135)	Baseline (*n* = 135)			Longitudinal (*n* = 93)		
		**Mild stroke** **Cluster I** **(*n* = 77)**	**Moderate stroke** **Cluster II** **(*n* = 58)**	***P*-value**^a^	**Mild stroke** **Cluster I** **(*n* = 51)**	**Moderate stroke** **Cluster II** **(*n* = 42)**	***P*-value**^a^
**Age**, years, mean (SD)	74 (12)	72 (12)	77 (13)	**0.01**	71 (13)	77 (10)	**0.03**
**Sex**, male/female (% female)	83/52 (38%)	47/30 (39%)	36/22 (38%)	0.9	31/20 (39%)	23/19 (45%)	0.14
**Stroke type**, ischemic infarct/ICH (% ischemic infarct)	125/9 (93%)	72/5 (94%)	53/4 (91%)	1.0	49/2 (96%)	39/3 (93%)	0.66
**Hemisphere of lesion **(n [%])				0.07			**0.02**
Left	34 (25%)	23 (30%)	11 (19%)		14 (28%)	8 (19%)	
Right	42 (31%)	26 (34%)	16 (28%)		18 (35%)	8 (19%)	
Bilateral	7 (5%)	3 (4%)	4 (7%)		1 (2%)	3 (7%)	
Cerebellum	10 (7%)	3 (4%)	7 (12%)		1 (2%)	6 (14%)	
Brain stem	3 (2%)	1 (1%)	2 (3%)		1 (2%)	1 (2%)	
Unclear	39 (29%)	21 (27%)	18 (31%)		16 (31%)	16 (38%)	
**OCSP classification** (n [%])^b^				0.52			0.87
TACS	5 (4%)	4 (5%)	1 (2%)		3 (6%)	0 (%)	
PACS	19 (14%)	9 (12%)	10 (17%)		6 (12%)	7 (17%)	
LACS	46 (34%)	30 (39%)	16 (28%)		19 (37%)	12 (29%)	
POCS	40 (30%)	20 (26%)	20 (35%)		15 (29%)	17 (41%)	
Unclear	13 (10%)	8 (10%)	5 (9%)		5 (10%)	2 (5%)	
**NIHSS**	2 (1–4)	2 (1–4)	2 (0–5)	0.79	2 (1–4)	2 (1–3)	0.98
**MoCA**	22 (19–25)	22 (19–26)	23 (19–26)	0.97	22 (19–24)	23 (19–25)	0.41
**BI-total score**	80 (65–90)	90 (85–95)	60 (55–70)	** < 0.001**	90 (85–95)	65 (55–75)	** < 0.001**
**mRS**	2 (1–4)	2 (1–2)	3 (2–3)	** < 0.001**	2 (1–2)	3 (2–3)	** < 0.001**
**TUG-time**	17 (8)	13 (4)	24 (9)	** < 0.001**	13 (4)	24 (9)	** < 0.001**
**HADS-A**	4 (1–8)	4 (0–7)	5 (2–9)	0.1	4 (0–7)	5 (2–8)	0.3
**HADS-D**	3 (1–6)	2 (0–6)	4 (1–7)	**0.02**	2 (0–7)	4 (1–7)	0.1
**FMA-UE motor function,** mean (SD)	59 (10)	60 (8)	57 (12)	0.11	60 (8)	57 (12)	0.23
**FMA-LE motor function,** mean (SD)	31 (4)	32 (3)	30 (5)	** < 0.001**	32 (3)	29 (6)	**0.001**
**BBS**^c^	49 (38–53)	52 (49–54)	38 (30–46)	** < 0.001**	52 (49–54)	39 (28–47)	** < 0.001**
Within 1 month					53 (50–55)	46 (40–50)	** < 0.001**
Within 2 months					54 (51–56)	48 (40–54)	** < 0.001**
3 months					54 (52–56)	49 (43–53)	** < 0.001**
1-year					54 (49–55)	49 (37–52)	** < 0.001**
***P*-value**^d^					** < 0.001**	**0.001**	

Data are presented without missing data imputation and give as median (25^th^ – 75^th^ percentile) unless otherwise noted. Significant values are indicated in bold

^a^Group comparison was conducted by using either Fisher’s exact test, Pearson χ^2^, Cochran-Armitage test, Mann–Whitney *U* test, or independent *t* test as appropriate, and *P*-values were determined

^b^Not applicable in patients with intracerebral haemorrhage

^c^Assistive devices: 1 patient was wheelchair dependent at baseline and was excluded in the longitudinal analysis. 2 patients used canes and 25 patients used walkers for assistance during the test

^d^The Friedman test was applied to analyze BBS scores from baseline to 1-year

*BBS* Berg Balance Scale, *BI* Barthel Index (baseline *n* = 134), *FMA* Fugl-Meyer Assessment (baseline *n* = 134), *HADS* Hospital Anxiety and Depression Scale (baseline *n* = 134), *NIHSS* National Institutes of Health Stroke Scale (baseline *n* = 99), *ICH* Intracerebral hemorrhage, *LE* Lower extremity, *MoCA* Montreal Cognitive Assessment (baseline *n* = 102), *mRS* modified Rankin Scale (baseline *n* = 128), *SD* Standard deviation, *TUG* Timed up-and-go test (baseline *n* = 126), *UE* Upper extremity, *OCSP* Oxford Community Stroke Project classification, *TACS* Total anterior circulation stroke, *PACS* Partial anterior circulation stroke, *LACS* Lacunar stroke, *POCS* Posterior circulation stroke

### Stroke severity based on baseline clustering

**Fig. 1:**
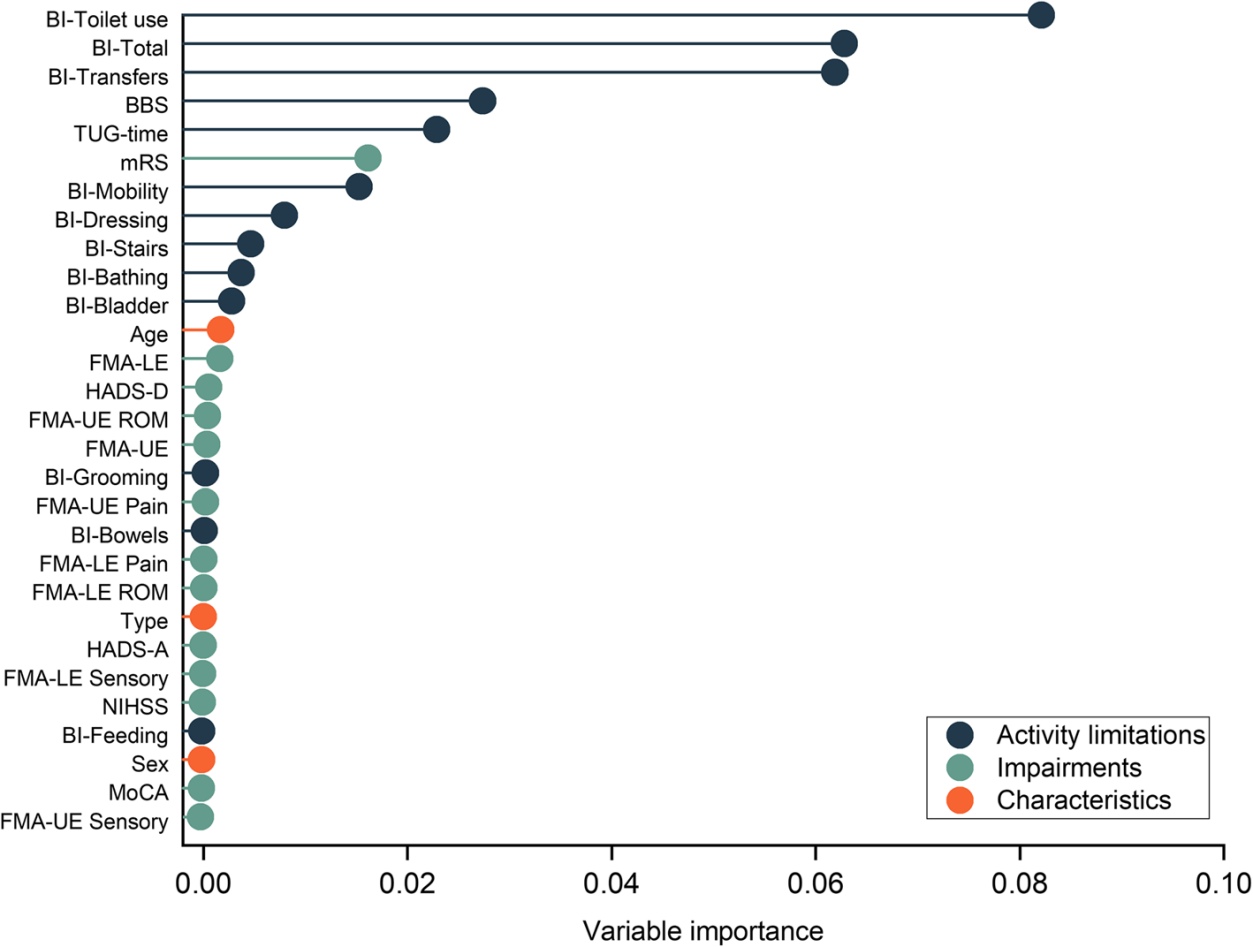
Variable importance of baseline variables in the cluster analysis. The corresponding domain of each variable is indicated following the framework of The International Classification of Functioning, Disability, and Health. The variable importance was derived from the mean square error with higher values indicating higher importance. Two clusters were determined as an optimal number of clusters on the basis of silhouette width (0.48). Stability of the clusters was assessed using the Jaccard similarity through resampling of the data 500 times. The Jaccard similarity was 0.97, which indicates stable clusters. BBS, Berg Balance Scale; BI, Barthel Index; FMA, Fugl-Meyer Assessment; HADS, Hospital Anxiety and Depression Scale; NIHSS, National Institutes of Health Stroke Scale; IQR, interquartile range; LE, lower extremity; MoCA, Montreal Cognitive Assessment; mRS, modified Rankin Scale; ROM, passive joint motion; SD, standard deviation; TUG, timed up-and-go test; UE, upper extremity

Using baseline clustering from 29 clinical variables, two distinct groups were stratified based on stroke severity by considering the overall impairment and activity limitations. Detailed clinical characteristics of the two clusters and variable importance for stratifying clusters are presented in Table 1 and Fig. 1.

The moderate affected stroke group included 58 patients (43%), and was characterized by a higher level of impairments and greater activity limitations which significantly differed from the mild groups (mean [SD] FMA-LE, 30 [5]; median [IQR] total BI scores, 60 [55–70]; median [IQR] mRS, mRS, 3 [2-3]).

The mild affected stroke group included 77 of the 135 patients (57%), and was characterized by mild impairments and slight activity limitations (mean [SD] FMA-LE, 32 [3]; median [IQR] total BI scores, 90 [85–95]; median [IQR] mRS, 2 [1-2]).

### Longitudinal changes in impairment of postural balance from baseline to 1-year

Ninety-three patients (54 [38%] mild stroke; 42 [62%] moderate stroke) with 636 assessments of BBS were included in the longitudinal analysis. Higher age (odds ratio [OR] 1.03, [95% CI, 1.02 to 1.05]) and worse cognition (OR 0.94 [95% CI, 0.9 to 0.98]) were significantly associated with greater impairment of postural balance, as presented in Table 2. Patients with moderate stroke (OR 3 [95% CI, 2.1 to 4.3]) had a significantly greater impaired postural balance, compared to patients with mild stroke.

**Table 2:** Longitudinal beta regression model for the proportion of balance impairments during the first year after stroke (*n* = 93)

	**Standardized β coefficient**	**Standard error**	**95% CI**	***P*-value**
**Intercept**	-3.5	0.8	-5.1 to -1.9	** < 0.001**
**Age**	0.03	0.008	0.016 to 0.05	** < 0.001**
**Cognition**	-0.06	0.02	-0.1 to -0.02	**0.005**
**Severity (mild stroke as reference)**				
Moderate	1.5	0.21	1.11 to 1.96	** < 0.001**
**Time (baseline as reference)**				
Within 1 mo	-0.16	0.13	-0.42 to 0.11	0.25
2 mos	-0.44	0.14	-0.72 to -0.17	**0.002**
3 mos	-0.5	0.14	-0.78 to -0.23	** < 0.001**
1-year	-0.18	0.13	-0.45 to 0.008	0.17
**Interaction- Severity × Time (Mild × baseline as reference)**				
Moderate × Within 1 mo	-0.51	0.17	-0.85 to -0.17	**0.004**
Moderate × 2 mos	-0.44	0.18	-0.79 to -0.1	**0.01**
Moderate × 3 mos	-0.61	0.18	-0.96 to -0.26	** < 0.001**
Moderate × 1-year	-0.65	0.17	-0.99 to -0.31	** < 0.001**

Significant values are indicated in bold. *CI* Confidence interval

**Fig. 2:**
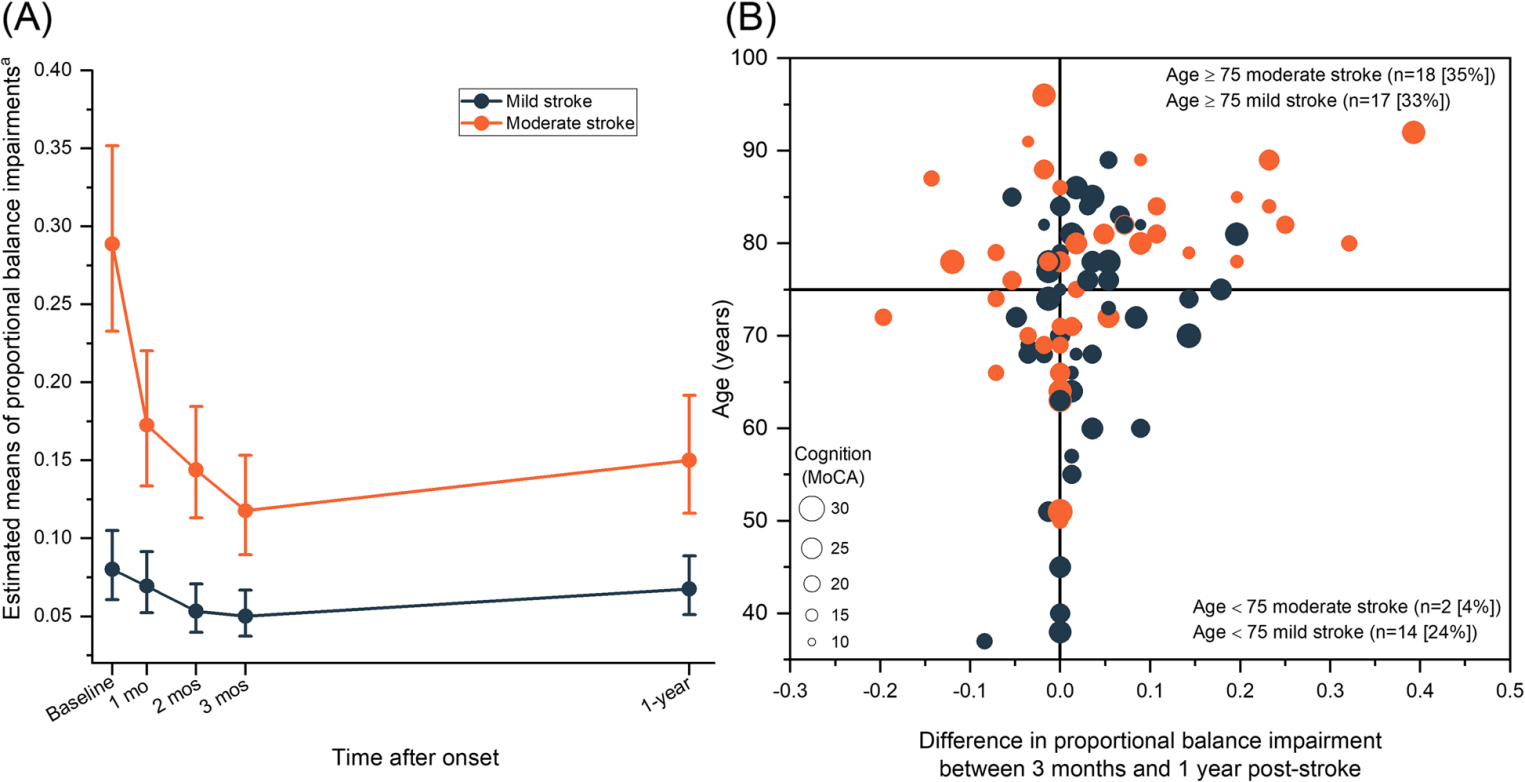
**A** Estimated means of proportional balance impairments and 95% confidence intervals across five time points by stroke severity. **B** Individual changes in proportional balance impairments by stroke severity, cognition, and age between 3 months and 1-year post-stroke. Difference ≥ 0 indicates an increase in balance impairment, whereas < 0 is a decrease in impairment. A total of 35 patients ≥ 75 years of age had an increase in balance impairment from 3 months to 1-year post-stroke. ^a^ Estimated means were converted from least square means after adjusting for age and cognition. MoCA, Montreal Cognitive Assessment

After adjusting for age and cognition, patients with moderate stroke had significantly improved from baseline to 1-year post-stroke, with BBS improving a median of 10 points (least-squares [LS] mean difference -0.83 [95% CI, -1.04 to -0.62]; adjusted *P* < 0.001). A reduction of 48% in the estimated mean proportional impairment of postural balance was found at 1-year for the moderate stroke group, compared to baseline (Fig. 2). For the mild affected stroke group, a decrease of 16% in the estimated mean was found from baseline to 1-year, but it was not statistically significant (LS mean difference, -0.18 [95% CI, -0.45 to 0.08], adjusted *P* = 0.34).

Both stroke severity groups had a maximum recovery at 3 months (LS mean difference, -1.11 [95% CI, -1.33 to -0.89], adjusted *P* < 0.001 for moderate stroke; and -0.5 [95% CI, -0.78 to -0.23], adjusted *P* = 0.002 for mild stroke, Fig. 2). A higher percentage of recovery was found in the moderate affected stroke group at 3 months (59% decrease in the estimated mean) compared to the mild stroke group (38% decrease).

### Changes in postural balance from 3 months to 1-year

Impairment of postural balance significantly increased from 3 months to 1-year in patients with moderate stroke, after adjusting for age and cognition (LS mean difference, 0.28 [95% CI, 0.05 to 0.51]; adjusted *P* = 0.015, Fig. 2). The increase in the estimated mean of proportional impairment of postural balance was 27% at 1-year after stroke, compared to 3 months.

For the mild affected stroke group, there was also an increase in impairment from 3 months to 1-year, but it was not statistically significant (35% increase in the estimated mean, LS mean difference 0.32 [95% CI, -0.02 to 0.66]; adjusted *P* = 0.07, Fig. 2).

A total of 51 of the 93 patients (55%) had an increased impairment of postural balance after 3 months. Individual differences in proportional impairment of postural balance between 3 months and 1-year post-stroke by stroke severity, age and cognition are shown in Fig. 2. Of these 51 patients with increased impairments, 35 patients (69%) were aged above 75 years old.

### Individuals with a BBS score below 45 during the first year of stroke

**Fig. 3:**
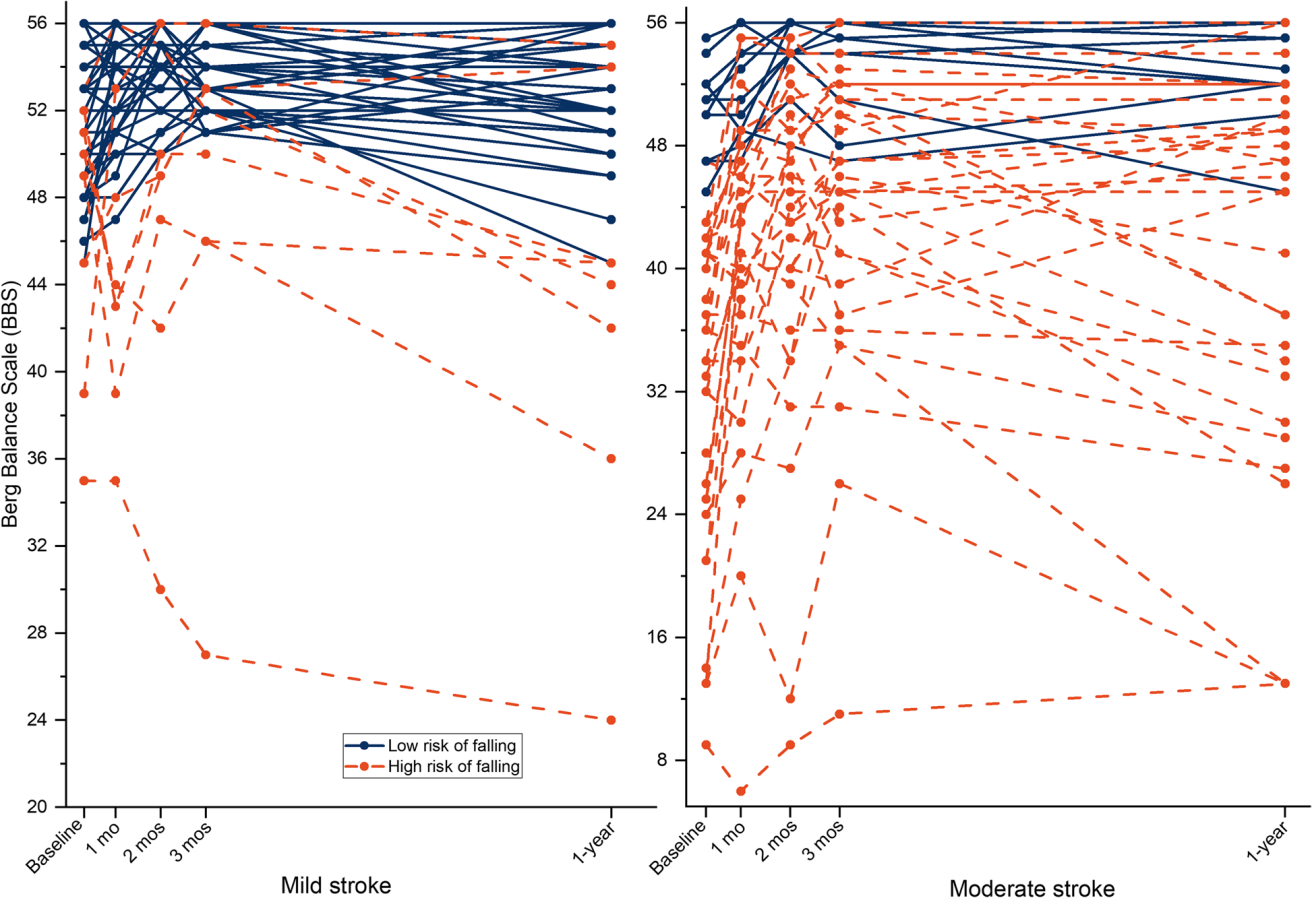
Longitudinal Berg Balance scale (BBS) in each individual by risk of falling in mild and moderate stroke. A cut-off of < 45 points, across any time point during the first year indicates individuals with a high risk of falling

**Fig. 4:**
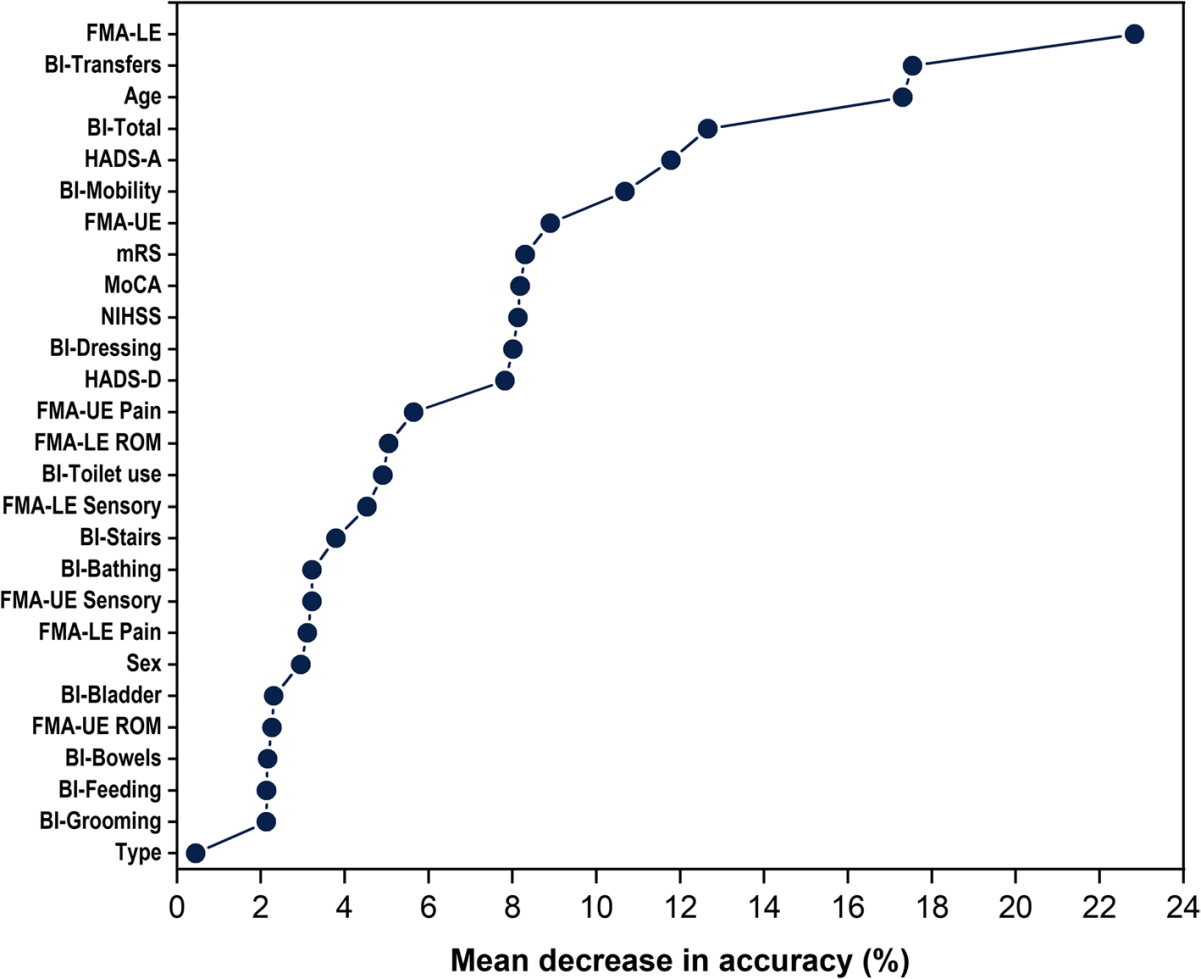
Mean decrease in accuracy after permutation of each variable in the random forest model. FMA-LE, BI-transfers, and age are the three most contributing variables for the model performance.BI, Barthel Index; FMA, Fugl-Meyer Assessment; HADS, Hospital Anxiety and Depression Scale; NIHSS, National Institutes of Health Stroke Scale; IQR, interquartile range; LE, lower extremity; MoCA, Montreal Cognitive Assessment; mRS, modified Rankin Scale; ROM, passive joint motion; SD, standard deviation; UE, upper extremity

Thirty-nine of the 93 patients (42%) were identified as having a BBS score < 45. Among these patients, 31 patients (79%) had moderate stroke and 8 (21%) had mild stroke. Longitudinal progression of balance in each individual with BBS < 45 or ≥ 45, by stroke severity across different time points, are presented in Fig. 3. FMA-LE, BI-transfers and age were three most contributing predictors for classifying patients who had BBS < 45 points within any time points of the first year after stroke (Fig. 4). Demographics and clinical variables between groups with BBS < 45 or ≥ 45 are presented in Table 3. The random forest model for classification, based on baseline predictors, achieved an accuracy of 0.98 (95% CI, 0.96 to 0.99), a sensitivity of 0.95 (95% CI, 0.91 to 0.98), a specificity of 0.99 (95% CI, 0.98 to 1), after tuning parameters with cross-validation.

**Table 3:** Demographics of patients with high or low risk of falling and 10 of the most predictive variables

**Characteristic**	**BBS < 45 (*n* = 39)**	**BBS ≥ 45 (*n* = 54)**	***P*-value**^a^
**Age**, years, mean (SD)	78 (10)	71 (12)	**0.006**
**Sex**, male/female (% female)	19/20 (51%)	35/19 (35%)	0.12
**Stroke type**, ischemic infarct/ICH (% ischemic infarct)	35/4 (90%)	53/1 (98%)	0.16
**Stroke severity**, moderate/mild (% moderate)	31/8 (79%)	31/11 (57%)	** < 0.001**
**FMA-UE motor function,** mean (SD)	56 (13)	61 (7)	0.16
**FMA-LE motor function,** mean (SD)	29 (6)	33 (2)	** < 0.001**
**BI total score**	70 (55–85)	85 (75–95)	** < 0.001**
**BI-Transfers,** n (%)			** < 0.001**
Major help	6 (15%)	0 (0%)	
Minor help	17 (44%)	10 (19%)	
Independent	16 (41%)	44 (81%)	
**BI-Mobility,** n (%)			** < 0.001**
Immobile/Major help	7 (18%)	3 (6%)	
Minor help	29 (74%)	25 (46%)	
Independent	3 (8%)	26 (48%)	
**NIHSS**	2 (1–3)	2 (1–4)	0.79
**MoCA**	20 (19–24)	23 (20–25)	0.05
**mRS**	3 (2–3)	2 (2–2)	** < 0.001**
**HADS-A**	4 (2–7)	4 (0–8)	0.6

Data are given as median (25^th^ – 75^th^ percentile) unless otherwise noted

*BI* Barthel Index, *FMA* Fugl-Meyer Assessment, *HADS* Hospital Anxiety and Depression Scale, *NIHSS* National Institutes of Health Stroke Scale, *ICH* Intracerebral hemorrhage, *LE* Lower extremity, *MoCA* Montreal Cognitive Assessment, *mRS* modified Rankin Scale, *SD* Standard deviation, *UE* Upper extremity

^a^*P*-values were determined by either Fisher’s exact test, Pearson χ^2^, Cochran-Armitage test, Mann–Whitney *U* test, or independent *t* test as appropriate. Significant values are indicated in bold

## Discussion

The study used multidimensional baseline measures to stratify stroke severity and examined the longitudinal progression in postural balance across each severity group. The main findings were that patients with moderate stroke had a significant recovery from their initial impaired postural balance assessed with BBS from baseline to 1-year, after adjusting for age and cognition. Both mild and moderate stroke showed a maximum recovery during the first 3 months post-stroke, and the patients with moderate stroke had significantly increased in their impairments of postural balance thereafter. Higher age and worse cognition were associated with more severe balance impairments. The baseline measurements showed a high sensitivity and specificity for classifying postural balance in patients that entails a potential risk of falling during the first-year post-stroke.

An increase of 10 scores in the median BBS from baseline to 1-year was relatively large, and was considered to be a minimal clinically important difference, as a reference of 6 points was suggested previously [33]. A recovery in impairment of postural balance during the first year post-stroke was expected, as an improvement in BBS was also demonstrated in prior studies at the 1-year follow-up post-stroke [34, 35]. However, in the present study, the significant recovery from the initial balance impairment (48%) was found only in patients with moderate stroke at 1-year, and it was not statistically significant for mild stroke. This was in line with earlier findings that only patients with more severe initial impairment significantly improved during the first year [8]. In addition, as demonstrated in earlier studies [36, 37], the ceiling effect in BBS may have impact on the ability to detect the potential improvement for patients with mild stroke, as they have relatively mild functional impairments.

The continuous recovery of impairment in postural balance seems to end at 3 months after onset for both moderate and mild stroke, assessed with BBS in the present study. This finding suggests that longitudinal functional recovery in stroke is similar in general, and may indicate similarities in the underlying mechanisms in the recovery of balance, as well as in other motor recoveries [7, 38]. The mechanisms underlying recovery after 3 months remain unclear, but could be a consequence of the diminished spontaneous recovery and ended the effect of rehabilitation [6]. However, we acknowledge that recovery might go on beyond the first year, although BBS probably not is the correct tool to assess this.

More severe initial impairments at baseline may be susceptible to deterioration after 3 months, as a statistically significant increase in impairments was found in only the moderate stroke group. Furthermore, older patients may recover less after 3 months. This was evident by a majority of patients aged ≥ 75 years (69%) experienced an increase in balance impairments, which has also been demonstrated in post-stroke functional mobility [8]. The current findings suggest that more frequent follow-up with physiotherapists and occupational therapists for patients with more severe stroke after 3 months may desirable to recognize the potential decline in balance function. This would help to early identify the needs of walking aids in individuals with stroke, and thus to prevent fall injuries.

In line with earlier findings [4, 39], worse cognition was associated with greater impairment of postural balance. However, cognition previously has been identified as a non-significant factor in post-stroke functional mobility [8]. The dissimilar impact of cognition may be attributable to the fact that the BBS assesses also static balance, which seems to require a higher cognitive input than solely walking does, with the ability to concentrate on holding body positions. This was further supported by the fact that greater functional connectivity between sensorimotor cortical areas showed in static balance than in the walking test, which suggests a greater cognitive impact required for static balance [40].

Even though baseline clustering based on a wide-ranging variable (e.g., impairments and activity limitations) was sufficient for handling a heterogeneity among stroke patients, a proportion of patients with moderate stroke had impaired balance along with good motor-sensory function in the extremities. This may be associated with potential involvement of a cerebellar lesion in some patients. Static balance is a complex behavior that does not only involve motor-sensory function in the extremities, but also perception, cognition and biomechanical constraints [41]. The BBS moderately correlates with the FMA [42, 43] which, in turn, may explain the variation noted in the present study.

As expected, lower-limb function, ability to transfer and age at baseline predicts well in patients with a risk of falling. This finding in line with the recognized predictors for risk of falling in previous studies [44–46]. Greater impairment of lower-limb function at baseline may, therefore, be important for the recognition of individuals at a risk of falling during the first year post-stroke. Improvements in balance have previously been shown in several forms of task training [47]. More longitudinal studies are warranted to explore the effect of training on the recovery of balance.

Impairment of postural balance was considered to be the most prioritized research area by stroke survivors due to concerns regarding fall incidents and disability in daily activities [48]. Fall evaluation may be necessary to consider in individuals with a BBS score lower than 45 points. Although some earlier studies have attempted to demonstrate different cut-off values of BBS for indicating individuals with a high risk of falling, the sensitivity and specificity has varied greatly (from 65 to 80%) [34, 45]. The cut-off value of BBS below 45 remained well accepted in clinical practice for clinicians to alert patients about potential risks of falling. However, determining different optimal cut-off values for BBS was not in the scope of this study. The focus on the longitudinal changes in postural balance, to identify patients who may have BBS below a pre-existed clinical threshold, across any time points, results in to general a more comprehensive picture of the progression in postural balance by different stroke severities. Therefore, the interpretation of the study results was limited to a risk of falling based on a single instrument instead of an actual fall detection or prediction that are complex and require consideration of multiple factors (e.g. medications, visual problems, structural barriers and environmental factors). This is not within the current aim and design of the study.

One strength of the study is the inclusion of multidimensional baseline measures (e.g. motor, cognition and psychological variables), this allowed clustering to classify stroke severity based on comprehensive clinical variables from impairments and activity limitations. It also largely contributes to handle the complexity of balance through taking into multidimensional parameters into account at baseline. The advances in the applied longitudinal beta regression model also considered the nature of high ordinal levels of BBS ratings, which avoids a loss of clinical information by converting to dichotomous or continuous bounded outcomes. Furthermore, the use of a mixed-effects model across stroke severities enhances the clinical interpretation of progression impairment of postural balance by taking between and within individual variability into consideration.

There are some limitations in the present study. One limitation of is that the data was collected on the basis of a randomized controlled design for examining outcomes between very early supported discharge and usual care. However, there were no significant differences were demonstrated between control and intervention group in postural balance at any time points in an earlier study [49]. As this study aim was to explore longitudinal changes in postural balance in the whole study population, the advance of baseline clustering used in the present study were able to provide more comprehensive classification of stroke severity. We therefore believe that the original study design has very little effect on the present study. Another limitation of this study, however, is that few patients with severe stroke were included in the study sample; therefore, the generalizability may be limited. Although the selected models were able to adapt to this limitation in the data, more data on patients with different degrees of stroke severity are desirable to confirm the study findings.

## Conclusion

The longitudinal analysis of postural balance indicates that the continuous recovery ends at 3 months regardless of mild or moderate stroke severity. Patients with moderate stroke had a significant postural balance recovery during the first 3 months, and then significantly diminished thereafter. Higher age and worse cognition were associated with greater impairment of postural balance. Baseline predictors, including motor-sensory function of the lower extremities, age, and ability to transfer, can accurately classify individuals with a potential risk of falling during the first year post-stroke.

## Data Availability

Data may be available to researchers upon request, after review of secrecy (contact the author Katharina Stibrant Sunnerhagen ks.sunnerhagen@neuro.gu.se). According to the Swedish regulation (epn.se/en/start/regulations/), the permission to use data can only be according to application and approval from the ethical board.

## Abbreviations

BBS: Berg Balance Scale
mRS: Modified Rankin Scale
BI: Barthel Index
FMA: Fugl-Meyer Assessment
MoCA: Montreal Cognitive Assessment
HADS: Hospital Anxiety and Depression Scale
NIHSS: National Institutes of Health Stroke Scale
TUG: Timed up-and-go test
